# Consumption habits of pregnant women and implications for developmental biology: a survey of predominantly Hispanic women in California

**DOI:** 10.1186/1475-2891-12-91

**Published:** 2013-07-01

**Authors:** Sarah E Santiago, Grace H Park, Kelly J Huffman

**Affiliations:** 1 Department of Psychology, University of California, 900 University Avenue, Riverside, CA, 92521-0128, California

**Keywords:** Prenatal exposure, Maternal diet, Environmental toxins, Fetal health

## Abstract

**Background:**

Healthy post-pregnancy outcomes are contingent upon an informed regimen of prenatal care encouraging healthy maternal consumption habits. In this article, we describe aspects of maternal intake of food, drink, and medication in a population of predominantly Hispanic women in Southern California. Potential implications for unhealthy prenatal dietary choices are discussed.

**Methods:**

The Food, Beverage, and Medication Intake Questionnaire (FBMIQ) measures common practices of maternal consumption during pregnancy. The FBMIQ was administered to English and Spanish speaking pregnant and recently pregnant (36 weeks pregnant - 8 weeks post-partum) women over the age of 18 who were receiving care from a private medical group in Downey CA.

**Results:**

A total of 200 women completed the FBMIQ. Consumption habits of healthy foods and beverages, unhealthy foods, unhealthy beverages, and medication are characterized in this article. Data indicate widespread consumption of fresh fruit, meats, milk and juice and indicate most women used prenatal vitamin supplements. Studies in developmental neuroscience have shown that certain substances may cause teratogenic effects on the fetus when ingested by the mother during pregnancy. Those potentially harmful substances included in our study were Bisphenol-A (BPA), methylmercury, caffeine, alcohol and certain medications. Our results show that a proportion of the women surveyed in our study consumed BPA, methylmercury, caffeine, alcohol, and certain medications at varied levels during pregnancy. This represents an interesting finding and suggests a disconnect between scientific data and general recommendations provided to pregnant mothers by obstetricians.

**Conclusions:**

The results of our study demonstrate that a proportion of pregnant women consume substances that are potentially teratogenic and may impact the health and well being of the offspring. It is important to appraise healthy and unhealthy consumption habits in order to encourage healthy practices and alleviate future effects of preventable, toxin-induced developmental issues. Prenatal advising should discourage the consumption of dangerous foods, beverages, and medications that women commonly report eating during pregnancy.

## Background

Post-partum outcomes for mother and child are linked to maternal consumption habits during pregnancy [1,2]. Thus, it is imperative that pregnant women be informed of the risks and benefits of certain dietary practices. This is not a simple task, as many foods, beverages and medications carry unknown risks [3]. Additionally, most primary care physicians and obstetricians are not aware of the dietary and over-the-counter medication intake practices of their patients and thus lack the information needed to help guide them. Assessment of common practices of food, drink, and medication intake during pregnancy informs the direction of preventative practice and interventions benefiting populations of pregnant women and their offspring. As prenatal exposure to certain environmental toxins, many of which are found in common foods and beverages, can lead to developmental deficits and malformations, common consumption practices must be appraised in current samples of pregnant women. These types of behavioral assessments play a critical role in prevention of adverse developmental outcomes that are potentially linked to a mother’s intake of unhealthy substances in food, beverages and medications throughout pregnancy.

Studies investigating the impact of prenatal diet on the offspring date back to the 1920’s [4], and many deleterious effects of poor diet have been reported in the literature. Reduced maternal nutrition has been associated with hypertension and altered nephrogenesis in the offspring [5,6]. Additionally, prenatal deficiencies in vitamins D and E have been associated with increased incidences of respiratory difficulties, including wheezing and asthma, in the offspring [7], and maternal vitamin D deficiencies have been related to observed hyper-locomotion in the adult rat [8]. Maternal diets high in omega-3 fatty acids may reduce sensitivity to allergies in the offspring [9], whereas methyl-donor group (vitamin B12, folic acid, and choline) supplementation during gestation is associated with increased risk of allergic inflammation in the offspring [10]. Increased cation consumption (magnesium, potassium, and calcium) was inversely related to diastolic pressure in infants [11]. These studies demonstrate that a healthy balance of nutrients play an important role in normal developmental biology.

Prenatal exposure to environmental toxins profoundly affects the developmental biology of the fetus. It is believed that exposure to toxins during the prenatal period induces developmental changes in the brain that lead to abnormal cognitive and behavioral phenotypes. Prenatal exposures to illegal drugs of abuse, such as cocaine, heroin, and methamphetamines, have been shown to impact both the developing brain and behavior [12-14]. Similarly, exposures to legal substances that are more commonly used during pregnancy, such as alcohol and nicotine, have been shown to have both short- and long-term effects on the developing baby [15-18]. Our developmental neurobiology laboratory has created two separate animal models of prenatal alcohol and nicotine exposure with compelling results [17,18], suggesting their high-risk status during pregnancy. It is common practice for obstetricians to warn pregnant women of the dangers of illegal drug use while pregnant, but there is less agreement in the field as to the exact recommendation for nicotine, caffeine, alcohol and many over-the-counter (OTC) medications and prescribed drugs [19-21]. Specifically, obstetricians vary greatly in their recommendations of alcohol consumption during pregnancy with some noting that occasional light use is permitted and safe, while others suggest complete abstinence [22,23]. There are no strict guidelines in obstetrics as to whether drugs labeled by the Food and Drug Administration (FDA) as Pregnancy Category C (found to generate birth defects in animal models) are to be prescribed. Obstetricians often disagree on safe levels of caffeine consumption during pregnancy, despite its link to negative behavioral effects in the offspring [24]. Some physicians may not be aware of how pervasive intake of legal substances such as alcohol, nicotine, caffeine or OTC medication may be and, thus, fail to inform the patient of details regarding use of these common, legal substances. Behavioral assessments of consumption of these potential teratogens, in populations of pregnant woman, are thus imperative to effective preventative obstetric practice.

In addition to illegal and legal drugs, significant sources of dietary impact on the developing fetus are teratogenic substances present in unassuming ordinary foods. A wealth of new information regarding the health of our nation’s food supply has been a subject of great recent interest and emerging studies have illuminated the harmful effects of methylmercury [25] and PCBs (polychlorinated biphenyls) [26], two common neurotoxic seafood contaminants present in high levels in tuna and farm-raised salmon, in offspring exposed to the toxins during gestation. Additionally, Bisphenol-A (BPA) is a potentially teratogenic xenoestrogenic monomer found in the lining of cans used for food storage. Consequences of prenatal exposure to BPAs include behavioral and reproductive abnormalities [27,28], increases in susceptibility for later developing mammary cancer [29], and alterations in reproductive systems. Understanding the rate at which pregnant woman consume certain food items is particularly important, as the list of foods that contain potential or known teratogens is growing. Researchers must strive to identify if pregnant woman are consuming toxins, and if so, preventative instructional measures should be taken by prenatal care providers to teach pregnant woman about the dangers of foods containing substances like mercury, PCBs, or BPA.

The Hispanic population is at risk for higher fat, sodium and caloric intakes, particularly from dairy foods [30,31]. Additionally, acculturation in Hispanic populations has been thought to result in diets higher in fat and lower in fiber, with significantly lower intakes of protein, calcium, vitamin A, vitamin C, and folic acid [32]. Risk factors such as low socioeconomic status (SES), acculturation to US diet, and limited access to health care in Hispanic populations increase the importance of studying this population in respect to potential adverse outcomes of poor prenatal diet [33].

This article describes our findings in a sample of pregnant or perinatal women in Downey, California. We used anonymous survey measures to determine what foods, beverages, and medications pregnant women were consuming throughout gestation (Additional file 1). Our results highlight the pervasive consumption of teratogens, such as mercury and BPA, in our sample. We hope that information gleaned from our study will help inform prenatal care providers of the dangers and nature of certain consumptive habits among pregnant women.

## Materials and methods

### Questionnaire

The Food, Beverage, and Medication Intake Questionnaire (FBMIQ) survey was designed to collect data on the prevalence and pattern of maternal consumption habits. The FBMIQ takes approximately five minutes to complete and contains questions concerning demographic information (household income, ethnicity, and age) how often and during what times during the pregnancy subjects (1) ate certain foods (e.g. fresh fruit, meat, and fast foods), (2) drank certain beverages (e.g. regular coffee, beer, and juice), and (3) ingested prescription and over-the-counter medications during pregnancy. The FBMIQ is not a complete dietary assessment; it is designed to be a short survey reflective of consumption of certain commonly consumed, relevant items. Portion size and number of servings consumed were not assessed. Based on our laboratory’s work in a Fetal Alcohol Spectrum Disorders (FASD) mouse model, we were initially interested in patients’ alcohol consumption during pregnancy. However, we comprised a list of potential or known teratogens as well as healthy and unhealthy practices and used a combination of these to create the survey. Non-threatening wording and reverse coding were used to maximize subject well-being and ensure accuracy.

### Participants and procedure

Pregnant and recently pregnant women (36 weeks pregnant - 8 weeks post-partum) over the age of 18 were invited to complete the FBMIQ upon check in at the reception desk of obstetric and gynecology (OBGYN) offices of a private medical group in Downey, CA between December, 2011 and December, 2012. Participants received the FBMIQ from receptionists and were informed that it was an optional nutritional survey about the habits of pregnant women. After completing the survey, participants placed completed consent forms and surveys in a blank sealable envelope provided to ensure confidentiality and alleviate concerns about anonymity. Surveys in sealed envelopes were then returned to the front desk where the receptionists collected the completed surveys. Frequency statistics for the data were presented in tables and differences between groups of age, education, and income were analyzed using Pearson correlation coefficients and non-parametric Kruskal-Wallis Tests. All statistical analyses were obtained using SPSS version 17.0. This study was conducted in strict accordance with the protocol guidelines approved by the Human Research Review Board at the University of Riverside, CA.

## Results

### Demographics and participant information

Participants were English and Spanish speaking pregnant or recently pregnant women (36 weeks pregnant - 8 weeks post-partum) over the age of 18 who were receiving Obstetric care from a private medical group in of Downey CA. A total of 200 pregnant or recently pregnant women completed the FBMIQ. Most women (89.1%) were aged 35 or younger, with the greatest percentage (34%) of participants belonging to the 30-35 age group. The subject population was predominantly Hispanic (87.4%). The remainder self-identified as 4.7% African-American, 4.2% Asian/Pacific Islander, 2.1% White, and 0.5% Middle-eastern. Almost all of the women (95.8%) had obtained a high school degree. Women were most likely to have completed some college without obtaining a final degree (41.9% of respondents). Less than a third of the women (26.2%) possessed a college or post-graduate degree. Women were most likely to have a yearly income of $25,000 or less (35.1%). Over two thirds (69%) of women sampled had a yearly income of 50 k or less. Thirteen point four percent had an income of over $75,000 (Table 1).

**Table 1:** Demographic information of respondents

**Age ( *n =194)***	**% Preg women**	**Education *(n=191)***	**% Preg women**
18=20	7.7	Elementary	0.5
21-25	20.1	Middle school	3.7
26-29	27.3	High school	27.7
30=35	34	Some college	41.9
36-39	8.2	College degree	17.8
40+	2.6	Graduate degree	8.4
**Race ( *n *= *190 *)**	**% Preg women**	**Income ( *n=171 *)**	**% Preg women**
White	2.1	0-25 K	35.1
Hispanic	87.4	25,001 K-50 K	33.9
African American	4.7	50,001-75 K	17.5
Asian/Pacific Islander	4.2	75,001 K-100 K	11.1
Middle-eastern	0.5	100 K+	2.3
Other	1.1		

Sample characteristics of respondents. Values for Age, Education, Race, and Income are listed as percentages for number of pregnant women. (n = 171-194).

### Month of pregnancy confirmation

Nearly half (50.5%) of the women sampled confirmed their pregnancies in the first month of gestation. That number rose to 88% by the second month. The vast majority of women (97.9%) confirmed pregnancy sometime in the first trimester (data not shown).

### Foods beverages and medicinal intake during pregnancy

Participants were asked about food, beverage, or medication intake during their pregnancies. Specifically, they were asked to identify types of foods, beverages or medications consumed and were given the option to write in choices that were not explicitly listed in the survey (See Tables 2, 3, 4). Participants were also asked to report the frequency and trimester specificity of consumption (Table 5).

**Table 2:** Food consumption habits

**Food consumed**	**% Preg women**
**Any meat**	99.5
Chicken	98.5
Beef	84
Pork	52
**Any fish**	73.9
Tuna	52
Tilapia	34.2
Salmon	25.5
Other fish	19.9
**Fresh fruit**	100
Banana	95.4
Oranges	88.8
Apples	88.3
Other fresh fruit	40.3
**Any canned foods**	73.9
Canned fruits/veggies	52.3
Canned soup	41.6
Canned tuna	41.6
**Sugary desserts**	97.5
Ice cream	82.7
Baked desserts	70.1
Chocolate	65
Other desserts	3.6
**Fast foods**	96
Burger	85.2
French fries	77.9
Chicken products	53.6
Others fast foods	7.7

Number of women who reported consuming a given food during pregnancy. Values are listed as percentages for number of pregnant women. (n = 195-200).

**Table 3:** Beverage consumption habits

**Beverage consumed**	**% Preg women**
**Any water**	**100**
Bottled water	96
Home filtered water	36.9
Tap water	12.1
Other water	1.5
**Any milk**	**95**.**4**
Lowfat	79
Whole	20.9
Other milk	9.2
Organic	6.7
Skim	4.1
**Any juice**	**94**.**9**
Orange juice	76.8
Apple juice	69.1
Juice blends	41.2
Other juice	12.9
**Any caffeine**	**80**.**1**
Colas	60.2
Coffee	45.5
Tea	29.8
Other caffeinated beverages	2.1
Energy drinks	1.5
Decaffeinated beverages	22.5
**Any alcohol**	**5**.**8**
**Beer**	1.6
**Wine**	4.7
**Mixed drinks**	1.1
**Shots/liquor**	0.5

Number of women who reported consuming a given beverage during pregnancy. Values are listed as percentages for number of pregnant women. (n = 188-199).

**Table 4:** **Medication**/**vitamin consumption**

**Substance consumed**	**% Preg women**
**Over-the-counter meds**	45.3
Acetaminophen	38
Cough/cold meds	4.2
Ibuprofen	3.1
Aspirin	1.6
Decongestants	1.6
**Prescription meds**	84
Prenatal vitamins	83.4
Morning sickness medications	8.8
Pain medications	3.6
Antidepressants	0

Number of women who reported consuming vitamins or medications during pregnancy. Values are listed as percentages for number of pregnant women. (n = 192-194).

**Table 5:** Frequency and trimester of consumption

**Substance consumed**	**Frequency (displayed as % of women reporting intake)**					**Trimester (displayed as % of women reporting intake)**						
	**1-3 entire preg.**	**1-3/month**	**1-3/week**	**4-6/week**	**7+/week**	**1**	**2**	**3**	**1,2,3**	**1,2**	**2,3**	**1,3**
**Food**												
Meat	5.2	12.9	51.5	22.2	8.2	9.9	9.4	3.6	66.1	2.6	7.8	0.5
Fish	41.4	38.6	16.6	2.8	0.7	12.3	27.5	8.7	31.2	4.3	14.5	1.4
Fruit	2.6	3.6	28	34.7	31.1	6.3	4.2	1.6	77	3.1	7.3	0.5
Canned foods	28	31.5	28.7	9.8	2.1	16.5	11.5	5	48.9	6.5	10.1	1.4
Sugary desserts	10.6	39.2	37	10.1	3.2	8.1	14	9.7	53.2	2.2	12.9	0
Fast foods	19.1	47.5	25.7	5.5	2.2	8.9	15.1	10.1	43	7.8	15.1	0
**Beverage**												
Water	1.5	0.5	3.1	6.7	88.2	5.8	3.2	0.5	85.8	2.1	2.1	0.5
Milk	2.2	8.2	23	35.5	31.1	6	4.4	1.1	71.4	4.9	11.5	0.5
Juice	2.8	11.7	35.8	30.7	19	7.4	6.3	2.8	68.8	6.3	8	0.6
Caffeine	14	30.7	40.7	11.3	3.3	10.9	15.4	8.3	40.4	5.1	18.6	1.1
Beer/wine	88.9	11.1	0	0	0	50	30	10	0	10	0	0
Mixed drinks/liquor	100	0	0	0	0	100	0	0	0	0	0	0
**Medication**												
Over-the-counter	53.7	31.3	7.5	3	4.5	19.3	21.7	13.3	28.9	6	7.2	3.6
Prescription	7.1	5.3	1.8	8.8	76.1	10.9	2	2	72.8	6.1	4.1	2

Frequency of consumption for foods, beverages, and medications and with trimester for which frequency data is applicable. All values are percentages of women who reported intake of a given item. Frequency values are listed as percentages for number of women who reported consuming a given item 1-3 times during their entire pregnancy, 1-3 times per month, 1-3 times per week, 4-6 times per week, or 7 or more times per week (n = 10-194). Trimester values are listed as percentages for number of women who reported consuming a given item during the first, second, or third trimester only, all three trimesters, or two of the three trimesters. (n=10-200).

### Food consumption habits

**Figure 1:**
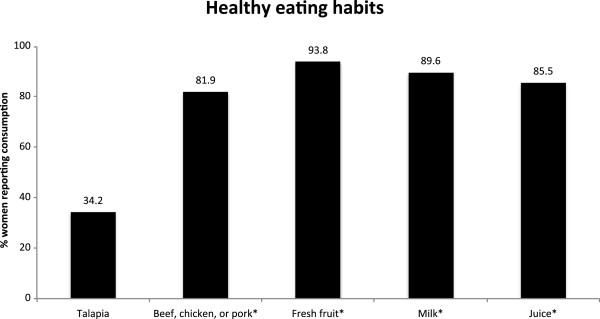
**Graphical depiction of percentages of women who reported eating healthy foods during their pregnancies.** Asterisks indicate percentages of women who ate or drank a given food or beverage at least once per week. (n = 196-200).

Participants were asked whether they ate chicken, beef, or pork during their pregnancies (fish consumption patterns were assessed in a different survey section, and thus fish was excluded from the meat category). Almost all of the women sampled (99.5%) ate meat some time during their pregnancies, and almost two-thirds (66.1%) ate meat during all three trimesters (Table 3, Table 5). Chicken was the most frequently reported meat consumed (98.5%), followed by beef (84%), then pork (52%). Most women (81.9%) consumed meat at least once a week, with 30.4% consuming meat at least four times a week (Figure 1, Table 2, Table 5).

Most participants (73.9%) consumed fish during pregnancy. The most commonly consumed type of fish was tuna (52%), followed by tilapia (34.2%) and salmon (25.5%). Less than a quarter of the women (19.9%) also reported eating other kinds of fish or shellfish, with shrimp being the most frequently written in option. Most women ate fish less than once a week (80%), and nearly a third of women (31.2%) ate fish during all three trimesters (Figure 1, Table 2, Table 5).

All women reported eating fresh fruit during their pregnancies. Bananas were the most commonly eaten fruit (95.4%), followed by oranges (88.8%), and apples (88.3%) (Figure 1, Table 2). Women also reported eating other fruits such as strawberries, pears, watermelon, and grapes. Two thirds (65.8%) of women ate fruit at least four times a week. Although the majority of women (77%) reported consuming fruit during all three trimesters of their pregnancies, only a third of the women (31.1%) ate the recommended amount of more than one serving of fruit per day (Table 5).

**Figure 2:**
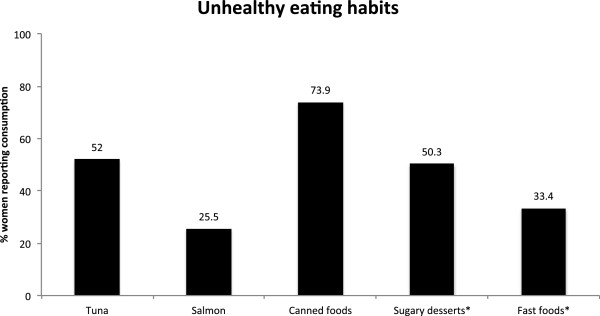
**Graphical depiction of percentages of women who reported eating unhealthy foods during their pregnancies.** Asterisks indicate percentages of women who ate a given food at least once per week. (n = 195 - 199).

The majority of participants (73.9%) reported consuming canned foods during their pregnancies, with 11.9% reporting consumption frequencies of four or more instances a week. Nearly half of sampled women (48.9%) reported eating canned foods during all three trimesters of their pregnancies (Table 5). Canned fruits/vegetables and soup were most commonly consumed (52.3% and 41.6% of women, respectively) followed by canned tuna (41.6%) (Figure 2; Table 2).

Almost all women (97.5%) reported eating high-sugar desserts during their pregnancies. Ice cream was the favored dessert: 82.7% reported eating ice cream. Additionally, 70.1% reported eating baked desserts, 65% reported eating chocolate, and 3.6% reported eating other desserts, such as candy or frozen dessert beverages during their pregnancies. Most women (76.2%) consumed desserts between one time per month and three times per week. Over half (53.2%) reported eating sugary desserts throughout their pregnancies (Figure 2, Table 2, Table 5).

Nearly all of the women (96%) reported eating fast foods during their pregnancies, with burgers being the most commonly consumed item (85.2%), followed by french fries (77.9%), chicken products (53.6%), and other fast foods such as Mexican fast foods and chicken salads. Consumption patterns for fast food intake were varied: 19.1% reported only eating fast foods 1-3 times during their entire pregnancies, 47.5% reported eating fast foods 1-3 times per month, and 25.7% reported eating fast foods 1-3 times per week. Only 5.5% of women reported eating fast food more than four times per week during their pregnancies. Forty-three percent of women reported eating fast foods during all three trimesters (Figure 2, Table 2, Table 5).

### Beverage consumption habits

All women reported drinking water, with 96% reporting consumption of bottled water, 36.9% consuming home-filtered water, and 12.1% consuming tap water during their pregnancies (Figures 1 and 2, Table 3).

Most women (95.4%) drank milk during their pregnancies. Of those women, 79% drank low-fat milk, 20.9% drank whole milk, 6.7% drank organic milk, 3.9% drank skim milk, and 8.6% drank “other” milk, with 3.6% identifying “other” as soy milk. Two-thirds of women (66.6%) drank milk at least 4 times per week. Most (71.4%) drank milk during all three trimesters. (Figure 1, Table 3, Table 5).

A total of 94.9% of women reported drinking juice during their pregnancies. Orange juice was the most commonly consumed juice (76.8%) followed by apple juice (69.1%), juice blends (41.2%) and other juices (12.9%) such as cranberry or pineapple juice. Most women (85.5%) reported drinking juice at least once per week, and over two thirds (68%) reported drinking juice throughout their pregnancies (Figure 1, Table 3, Table 5).

**Figure 3:**
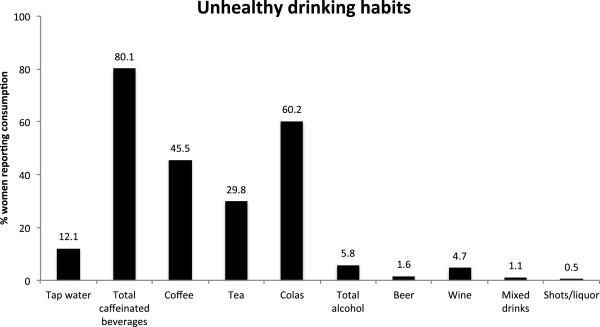
**Graphical depiction of percentages of women who reported drinking unhealthy beverages during their pregnancies.** (n = 188 - 199).

Participants were asked to report whether they had consumed regular coffee, regular tea, colas, or decaffeinated beverages during their pregnancies. The majority of women sampled (80.1%) had consumed caffeinated beverages during their pregnancies (Figure 3, Table 3). Colas were the most popular caffeinated beverage (60.2% of all pregnant women), followed by coffee (45.5%) and tea (29.8%). Forty point four percent of women who reported drinking caffeinated beverages did so throughout their pregnancies, while nearly a fifth (18.6%) only consumed caffeine during the mid- and later parts of their pregnancies (Figure 3, Table 3, Table 5).

Out of 190 women who responded to any questions about alcohol usage, 5.8% reported drinking alcohol sometime during their pregnancies (Figure 3, Table 3). When asked about the type of alcohol consumed, these women reported drinking beer, wine, or champagne. Consumption of mixed drinks and hard liquor was less common, with only a couple of women reporting this type of alcohol use (Figure 3, Table 3).

Number of alcohol units consumed during pregnancy was relatively low; most women (nine out of the ten who reported gestational alcohol use) only consumed 1 - 3 units of alcohol (bottles, glasses, or shots) during the entire pregnancy. The exception was one participant who reported an alcohol consumption rate of 1-3 alcohol units per month during the first trimester (Table 5).

Alcohol usage typically occurred during the first trimester, with the exception of four participants, who reported alcohol usage in the second or third trimester (Table 5).

### Consumption of vitamins and medications

Most women reported consuming prenatal vitamins during their pregnancies (83.4%). Eight point eight percent of women reported taking prescription morning sickness medicine, and 3.6% reported taking prescription pain medications. Acetaminophen was the highest used over-the-counter medication (38%). A small number of women reported taking aspirin (1.6%), ibuprofen (3.1%), decongestants (1.6%) and cough and cold medications (4.2%; Table 4) during their pregnancies.

### Demographic factors and risky consumption habits

Pearson correlation coefficients between demographic factors (age, education, and income) and dietary risk factors (consumption of tuna, salmon, canned foods, sugary desserts, fast foods, tap water, caffeine, and alcohol) were calculated. Women with lower income were more likely to have reported eating any canned foods during their pregnancies (r = -0.143; p <0.05; n = 191). No other significant correlations were found.

Non-parametric Kruskal-Wallis Tests were performed to explore differences in consumption frequencies for fish, canned foods, sugary desserts, fast foods, caffeinated beverages, and alcohol with grouping variables of age, education, and income. Kruskal-Wallis tests revealed no significant differences in consumption frequencies among groups.

## Discussion

### Healthy eating and drinking

Consumption of beef, chicken, tilapia, fruit, bottled or filtered water, milk, juice, and decaffeinated beverages were considered to be generally safe and healthy for the developing fetus. While recent risk factors associated with meat products have been described [34], the beneficial dietary supplementation of these choline-rich foods has been judged to outweigh the risks associated with hormone and antibiotic levels in certain meat and diary products. Likewise, high-sugar content in certain types of juices may be associated with the onset of gestational diabetes; however, the vitamins and nutrients in juices merit their inclusion in the healthy drinking category. The American Dietetic Association recommends, for pregnant women, a healthy diet in accordance with the Dietary Guidelines for Americans (2005) including a variety of daily grains, milk products, fruits, vegetables, and iron-rich meats [1,35]. The majority of pregnant women reported eating healthy foods including tilapia, beef, chicken, or pork, fresh fruit, milk, and juice during their pregnancies. The frequencies at which women in our sample ate these foods did not strictly adhere to daily recommendations. Only a third of women, for example, reported eating fruit 7 or more times a week.

### Unhealthy consumption habits

Consumption of tuna, salmon, canned goods, sugary desserts, fast foods, and drinking of tap water, caffeinated beverages, and alcoholic beverages during pregnancy have been deemed unhealthy due to the appearance of environmental toxins found to have harmful effects in the developing offspring.

### Tuna

The bioaccumulation of methylmercury (MeHg) in marine life, particularly tuna, presents a threat for developing fetuses whose mothers frequently eat this fish during their pregnancies, particularly because it is thought that mercury accumulates more readily in the fetal brain than in the maternal brain, interrupting patterns of cell fate, proliferation, migration, and neural outgrowth [36-38]. Because young children cannot metabolize mercury at the same rates as adults, exposure through either maternal or childhood consumption is of great concern. Even low levels of tuna consumption in children can readily result in blood mercury levels that exceed the heath limit [39]. Epidemiological studies with cohorts from fish-eating populations have found that prenatal exposure to methylmercury has been associated with a myriad of developmental deficits involving attention, verbal learning, visuo-spatial and motor function, and delayed performance [25,40]. The FDA reports that mean mercury levels of tuna range from 0.128 ppm (light canned tuna) to 0.689 ppm (big-eye tuna). However, mercury levels are highly variable and the FDA reported canned tuna to contain as much as 0.889 ppm for light canned tuna, and 1.816 ppm for big-eye tuna [41]. Currently, the FDA and the Environmental Protection Agency (EPA) recommend that pregnant women eat no more than six ounces of tuna per week. However, tests performed by Consumer Reports on new samples of white tuna revealed that eating only 2.5 ounces of any of the new samples of white tuna would cause pregnant women to exceed the daily mercury levels that the EPA considers safe [42]. Worryingly, more than half of the women (52%) in this study reported consuming tuna during their pregnancies, suggesting that pregnant woman are generally not aware of risk associated with tuna consumption.

### Salmon

Polychlorinated biphenyls (PCBs) are lipophilic compounds found in fatty tissues of marine life feeding in contaminated waters. Staggering levels of PCBs have been found in farmed salmon compared to their wild-type counterparts, and contaminated commercial salmon feed has lead to the bioaccumulation of these dangerous compounds [43]. Prenatal exposure of PCBs has been linked to lower birth weights, smaller head circumferences, and abnormal reflex abilities in newborns, as well as to mental impairment in older children [26,44-46]. The FDA limits limiting PCB residues in fish to 2 ppm [47]. Although epidemiological studies seem to suggest PCB exposure is related to poor outcomes in neurodevelopment, the precise exposure patterns leading to these deficits have not been characterized [46]. However, a recent meta-analysis of 12 European studies found an association between fetal growth and PCB exposure at low, clinically-relevant levels [48].

More than a quarter of the women (25.5%) surveyed reported consuming salmon, a commonly ingested source of PCBs. Although prenatal fish intake, including tuna and salmon, can be a good source of Docosahexaenoic acid (DHA), a fatty acid thought to be beneficial in development, fetuses may be at risk for adverse outcomes, and pregnant women should be advised to be selective about which fish they choose to consume, or seek supplementation with fish oil.

### Canned foods

Bisphenol A (BPA) found in the lining of metal cans used for food represents a danger to developing fetuses whose mothers consumed a diet high in canned foods. BPA has received recent attention as a controversial ingredient in child sippy cups, baby bottles, and reusable water bottles, leading to a ban by the FDA on the use of the plastic additive in sippy cups and baby bottles [49,50]. Perhaps less well-known are the dangers of BPA exposure in consuming canned foods. Leaching of BPA from the epoxy resin of metallic food cans has been demonstrated in many studies [49,51,52]. Prenatal exposure to BPA has been found to be associated with higher externalizing scores (hyperactivity and aggression) in two-year-old females and reproductive effects in rodent models [27,28]. The majority of women surveyed (73.9%) reported consumption of canned foods during their pregnancies, with 11.9% reporting consumption at least 4 times a week, suggesting women may not be aware of the dangers of BPA exposure from canned foods. Although epidemiological studies concerning prenatal BPA exposure are lacking, animal studies warn of the detrimental effects of BPAs. Children of pregnant women maintaining a diet high in canned foods are at risk for adverse postnatal outcomes. This study has found that low income is inversely correlated with canned food consumption, suggesting that women of low SES in particular may be especially at risk.

### Sweet desserts

Frequent consumption of sugary desserts during pregnancy may contribute to increased likely-hood of gestational diabetes mellitus (GDM), a condition of glucose intolerance that has been implicated in many pregnancy problems including macrosomia, large for gestational age (LGA) infants, and increased rates of cesarean delivery [53-57]. Hispanic women in particular are two and half times more likely than non-Hispanic whites to suffer from GDM [58]. Increased sugar intake among pregnant adolescents has been linked to maternal gestational diabetes and LGA infants [57,59,60], and problems with gestational glucose control have been associated with neural tube defects [61]. On the other hand, carbohydrate restriction has been shown to aid in maternal glycemic control, alleviating some of the adverse pregnancy outcomes seen in patients with GDM [54]. More than a third of women (37%) ate sweet desserts more than one time per week, and 13.3% ate desserts more than 4 times per week. Children born of women maintaining a diet high in sweet desserts are at risk of macrosomia and postnatal obesity. Of course, women with GDM should be advised to closely monitor their sugar intake during pregnancy.

### Fast foods

The past decades have seen an insurgence of reliance upon high-energy, low nutrient foods that correlate with rising rates of obesity. A prenatal diet high in fast food represents a possible danger to the developing fetus, as these foods usually contain high levels of fat and salt. Maternal diets high in fat have been associated with increased likelihoods of postnatal diet-induced obesity in offspring, and have the potential to influence epigenetic markers leading to altered postnatal gene expression and eating behavior [62,63], Prenatal diets high in sodium levels have been linked to decreased gestational weight gain and an increased responsiveness to stress in adults [64,65]. More than a quarter of women surveyed (25.7%) report eating fast foods at least once a week during their pregnancies. An alarmingly high percentage of surveyed women reported consuming fast foods more than four times per week (7.7%), and are at heightened risk for adverse fetal effects of high maternal salt and fat diets. Additionally, Hispanic populations are at risk for higher fat intakes from dairy foods [30]. Based on the literature, prenatal advising should stress the importance of eating a healthy diet low in these energy-dense, nutrient low foods so as to lower future generations’ risk of obesity and stress conditions.

### Tap water

Drinking water has been found to have levels of many prenatal toxins including trihalomethanes, and certain drinking water disinfection by-products (dibromoacetic acids, or DBAs). The Drinking Water Quality Report for Downey, CA warns of 20 different pollutants in the city’s water, with eight chemicals existing at concentrations that exceed the health guidelines set by federal and state agencies: tri - and tetro-chloroethylene (DBAs), alpha particle activity, arsenic, radium 228, lead, radium 226, and combined radium [66]. Many women in our sample (12.1%) reported drinking tap water during pregnancy, suggesting risk for exposure to many of these dangerous contaminants. Contaminants such as arsenic and DBA, and radium 226 have been found to result in central nervous system defects, oral cleft defects, and neural tube defects, and small for gestational age births, and risks for fetal death [67-69]. Women drinking tap water in areas where contamination is exceptionally high, as in Downey, CA, are at risk for adverse outcomes resulting from prenatal exposure to certain chemicals. Pregnant women should instead be encouraged to drink filtered or bottled water.

### Alcohol

The spectrum of disabilities associated with prenatal alcohol exposure is termed FASD (Fetal Alcohol Spectrum Disorders). The prevalence of FASD has been difficult for researchers to ascertain. Some studies have found that anywhere from 0.5 - 2 cases per live births [70]. One study found as high as 1 per 100 live births when the full range of FASD was taken into account [71]. There is a large amount of data characterizing the severe complications in children who were exposed prenatally to alcohol. Children born with FASD often exhibit abnormal craniofacial features and have a litany of cognitive impairments including learning disabilities, decreased intelligence, decreased reaction time, slow sensory processing speed, language dysfunction, and behavioral disorders that are direct results of nervous system injury [72-75].

### Self-reporting of maternal drinking

Information on the prevalence and pattern of maternal drinking has been notoriously difficult to ascertain. One possible diagnosis tool is the detection of biomarkers at birth. FAEEs (fatty acid ethyl esters) accumulate in the meconium after alcohol exposure in the first and second trimester [76]. Such methods have been useful in detecting alcohol usage in at-risk subjects at birth, but are impractical for use in a comprehensive assessment of average alcohol usage during the entire gestational period. Thus, although biological methods of measuring alcohol usage during pregnancy have been developed, self-reports of maternal drinking remain the most effective methodology for maternal drinking prevalence assessments.

Ten women (5.8%) surveyed in Downey, CA reported drinking some time during their pregnancies. This number is slightly lower than national estimates of maternal drinking from the Centers for Disease Control and Prevention (CDC; 7.6% of pregnant women surveyed) [77]. Differences in rates may be due to the demographic make up of our sample. Maternal drinking rates are highest in white populations of older individuals (35-44 years of age) [77]. This study examined a population of relatively young (89.2% under 35) women who were predominantly Hispanic (87.4%). Methodological differences may also be at play. CDC data is obtained from the Behavioral Risk Factor Surveillance System (BRFSS), a telephone survey system that asks women to report alcohol usage within the last 30 days. Conversely, the current study surveys participants in late pregnancy or early post-pregnancy periods, when subjects might have difficulty remembering nutritional habits from early pregnancy. The chance of reporting inaccurate information from early pregnancy rises with the passing of time, and the stigma associated with maternal drinking might influence participants to deny usage if recall was suspect. However, despite the difficulties in obtaining accurate data through self-report, administration of a survey instrument to this population of women is the best way to obtain information regarding behaviors during pregnancy.

### Methyl donors, prenatal vitamins, and alcohol consumption

Research has consistently shown that intake of folate, choline, and other methyl donors are integral to the healthy development of the fetus. Specifically, the metabolism of these nutrients provides methyl groups in one-carbon methylation pathways [78]. Disruptions in one-carbon metabolism may result in decreased cognitive abilities [79] and serious birth defects [80]. Folic acid is an important contributor of methyl groups for pregnant women. A recent study has suggested that intake of prenatal vitamins may reduce the risk of autism [81]. Fortunately, intake of prenatal vitamins is fairly common: 83.4% of women report supplementing their diets with prenatal vitamins. Additionally, consumption of choline-rich foods was high: all women reported eating meat sometime during their pregnancies, and most reported eating meat at least once per week.

Because prenatal alcohol has been found to disrupt one-carbon metabolism, diets deficient in methyl donors may exacerbate the harmful effects of prenatal alcohol. Although women seem to be making an effort to curb their alcohol usage after recognizing their pregnancy, the number of women who report drinking during the first trimester is worrying (half of the ten women who reported drinking). Research has shown early pregnancy to be especially vulnerable to the teratogenic effects of alcohol, as deficiencies in methyl donor groups in the first month have been shown to result in neural tube birth defects in offspring [78].

### Caffeine usage

Caffeine is a xanthine alkaloid that can be found in coffee, sodas, energy drinks, and tea. Studies labeling caffeine as a teratogen date back to the late 1960s [82], and in 1980, the FDA advised limiting intake of caffeine during pregnancy, noting the substance’s association with fetal mortality, birth defects, and decreased birth weights [83,84]. The American Pregnancy Association recommends 150-300 mg as a safe daily dose of caffeine, although this is only based upon studies concerning risk of miscarriage [85]. Published epidemiological studies noting the impact of prenatal caffeine on child behavioral and cognitive effects have been somewhat scarce and inconclusive [24,86-88]. Animal studies, however, have found developmental delays, abnormal neuro-motor activity, and neurochemical disruptions, with some effects persisting until adulthood (for review, [89]). High levels of coffee consumption have been linked to fetal death after the second trimester [90,91].

Caffeine has been consistently linked to abnormal motor activity and motor development [87,88]. A majority of the subjects (80%) reported drinking caffeinated beverages during their pregnancies, with 14% of women reporting consumption of more than 4 caffeinated beverages per week. These numbers suggest that some children may be at risk for preventable persistent aberrations in neurochemistry and motor development.

### Intake of over-the-counter and prescription medication

The number of women taking over-the-counter and prescription medications during pregnancy has increased within the past few decades [92]. A small number of women report using aspirin (1.6%) and ibuprofen (3.1%) during pregnancy. Animal experiments have implicated aspirin and ibuprofen, both cyclooxygenase inhibitors, in a number of adverse fetal effects including physical malformations [93,94] and postnatal cognitive deficits [95].

A small number of women (3.6%) reported taking prescription pain medications during pregnancy. Fetal effects as a result of prenatal opioid exposure are poorly understood, but seem to be related to poor developmental outcomes [96]. Case control studies have made associations between prenatal use of opioid analgesics and congenital heart defects, the primary factor in birth-defect related infant mortalities [97].

Less than 10% (8.8%) of women reported using prescription anti-nausea medications. Use of anti-nausea medications have been found to be associated with acute non-lymphoblastic leukemia [98] although some FDA pregnancy category B drugs (considered safe in pregnancy) such as Zofran, are often prescribed for pregnancy related morning sickness. Our study did not differentiate between the pregnancy categories of anti-nausea medications used.

A paucity of conclusive research on the effects of prenatal exposure to prescription drugs may have led to the assumption that they are safe to prescribe. However, studies highlighting the possible dangers of prescription drugs, as well as some over-the-counter medications, suggest otherwise. Use of opioid analgesics and category C and above anti-nausea medications put pregnant women at risk for aversive offspring outcomes. In any case, women should be properly informed as to the possible dangers of prenatal exposure to these medications.

### Changing consumption patterns after recognition of pregnancy

Reports of patterns of consumption, that is, information about the amount of a substance consumed and the period during pregnancy in which it was consumed, can provide a window into commonplace beliefs about what habits are healthy during gestation. For example, most women who consumed alcohol during pregnancy reported doing so only in the first trimester. Given that a significant number of women (48.6%) confirm their pregnancies halfway through the trimester, it is likely that these women who report drinking during the first few months are doing so before realizing they are pregnant. In contrast, the majority of women consuming caffeine during pregnancy report continuing their consumption of these beverages well into their second and third trimesters, suggesting that caffeine is not commonly regarded as harmful to the unborn fetus. The implications of this research are two fold: firstly, women of childbearing age hoping to conceive should be advised to eliminate all alcohol consumption, as effects of maternal drinking have dire consequences in the first trimester when the mother may not know she is pregnant. Additionally, every effort should be made during clinical prenatal care visits to inform pregnant women of the harmful effects of environmental toxins that can be readily transferred to the fetus as a result of uninformed, unhealthy consumption habits.

### Study limitations

Several problems exist in attempt to extract such sensitive data (such as gestational consumption of alcohol) from participants, with underreporting and recall bias as major hurdles to accurate data collection. The uncomfortable nature of questions concerning maternal drinking may lead to extensive underreporting of alcohol consumption. Underreporting is suspected to be a major issue in the field [99]. Nevertheless, self-reports of maternal drinking remain the most effective tool for sensitive assessments.

Unfortunately, our data was limited to women who agreed to participate in our survey, and we were unable to randomize samples. As survey distribution was offsite at a private medical group in Downey, CA, we do not have information on percentages of women who were not included in the survey. Small sample sizes for other ethnicities such as Whites, African Americans, and Asians made cross-ethnic studies unfeasible. Similarly, we were unable to conduct certain analyses on a low but significant population of women reporting alcohol use during pregnancy. The FBMIQ was designed to be a short 5-minute study assessing percentages of women who may be at risk for certain unsafe consumption habits and as such it does not represent a complete dietary intake assessment. The scope of the survey limited us to frequency and trimester information only in major food categories, thus we were unable to report frequencies of subcategory items (prenatal vitamins versus prescription pain medication, for example). Additionally,although many substances in foods and beverages are thought to be teratogenic at high levels, specific dosages and patterns of exposure leading to adverse outcomes are not yet known. Developmental deficits as a result of prenatal exposure to environmental toxins at clinically-relevant levels, along with reviews and meta-analyses of the current literature, are important areas of future research that will help inform future prenatal guidance protocols.

## Conclusions

The current study analyzes dietary habits of pregnant or recently pregnant women in Downey, CA, with particular emphasis on consumption intake of substances thought to be teratogenic in nature. Our main findings are summaries as percentages of women reporting consumption of unhealthy foods and beverages during pregnancies. For example, we found high numbers of Hispanic pregnant women consumed methylmercury through tuna, PCBs through salmon consumption, BPA through canned goods, DBAs containing tap water, caffeine containing beverages, and alcohol containing beverages during pregnancy. We also found that large percentages of pregnant Hispanic women reported eating high sugar sweet desserts and high fat and salt fast foods more than once a week. A small number of women reported the use of certain non-recommended over-the-counter medications such as aspirin and ibuprofen, as well as prescription medications with unsafe FDA pregnancy categories. These data reflect a remaining risk in certain populations for adverse outcomes in fetal development. Fortunately, a majority of the women surveyed report taking prenatal vitamins, which aid in the prevention of many neural tube defects. Additionally, percentages women reporting healthy consumption habits were generally high. In summary, our findings in a population of predominantly Hispanic women suggest high levels of consumption of substances that serve as potential teratogens to unborn children. Because we have not surveyed other populations of pregnant women, we do not know whether this is something unique to Hispanic women, or ubiquitous among women of multiple ethnicities. However, it is clear that prenatal medical professionals should discourage the consumption of dangerous foods, beverages, and medications that women commonly report consuming during pregnancy. In light of our consumption data here, prenatal professionals, including but not limited to OBGYNs, should be encouraged to instruct their pregnant patients about the dangers of hidden teratogens in our food supply, including but not limited to methylmercury and PCBs in fish, caffeine and alcohol in beverages and BPA in canned goods.

## Competing interest

The authors declare that they have no competing interest.

## Authors’ contributions

SS participated in the coordination of the study, performed the statistical analysis, helped draft the format of the survey, and helped draft the manuscript, figures, and tables. GP helped conceive the initial design of the study and contributed to the creation of the original draft of the survey. KH designed the study, participated in coordination of the study, and helped draft the survey and finalized the manuscript, figures and tables. All authors read and approved the final manuscript.

## Supplementary Material

Additional file 1 Anonymous (no names) survey. Click here for file
