# Supplementary material for: Consumption habits of pregnant women and implications for developmental biology: a survey of predominantly Hispanic women in California
Source: Nutr J. 2013 Jul 1;12:91. doi: 10.1186/1475-2891-12-91 (PMC3704911; doi:10.1186/1475-2891-12-91)
Supplement: Additional file 1 — Anonymous (no names) survey. [file 1475-2891-12-91-S1.pdf]

ANONYMOUS (NO NAMES) SURVEY

Thank you for participating in this anonymous survey. This survey is a part of a study at University of California, Riverside on the nutritional (eating and drinking) habits of pregnant women. We would like to know what foods, drinks and medications you took during your pregnancy, during approximately what time period or trimester.

For each question please check ALL that apply. More than one box may be checked.

During your pregnancy, did you eat…			
	**What kind?**	**How often?**	**When?**
**1. Meat?**<br><br>☐ **Yes**<br>☐ **No**	☐ chicken<br>☐ beef<br>☐ pork<br>☐ other:<br>__________	☐ 1-3 times in entire pregnancy<br>☐ 1-3 times per month<br>☐ 1-3 times per week<br>☐ 4-6 times per week<br>☐ 7+ times per week	☐ beginning of pregnancy<br>☐ middle of pregnancy<br>☐ end of pregnancy
**2. Fish?**<br><br>☐ **Yes**<br>☐ **No**	☐ tuna<br>☐ salmon<br>☐ talapia<br>☐ other:<br>__________	☐ 1-3 times in entire pregnancy<br>☐ 1-3 times per month<br>☐ 1-3 times per week<br>☐ 4-6 times per week<br>☐ 7+ times per week	☐ beginning of pregnancy<br>☐ middle of pregnancy<br>☐ end of pregnancy
**3. Canned Foods?**<br><br>☐ **Yes**<br>☐ **No**	☐ fruit or vegetables<br>☐ soup<br>☐ tuna<br>☐ other: ___________	☐ 1-3 times in entire pregnancy<br>☐ 1-3 times per month<br>☐ 1-3 times per week<br>☐ 4-6 times per week<br>☐ 7+ times per week	☐ beginning of pregnancy<br>☐ middle of pregnancy<br>☐ end of pregnancy
**4. Sugary Deserts?**<br><br>☐ **Yes**<br>☐ **No**	☐ chocolate<br>☐ ice cream<br>☐ baked goods<br>☐ other:<br>__________	☐ 1-3 times in entire pregnancy<br>☐ 1-3 times per month<br>☐ 1-3 times per week<br>☐ 4-6 times per week<br>☐ 7+ times per week	☐ beginning of pregnancy<br>☐ middle of pregnancy<br>☐ end of pregnancy
**5. Fast Foods?**<br><br>☐ **Yes**<br>☐ **No**	☐ burgers<br>☐ chicken products<br>☐ french fries<br>☐ other:<br>__________	☐ 1-3 times in entire pregnancy<br>☐ 1-3 times per month<br>☐ 1-3 times per week<br>☐ 4-6 times per week<br>☐ 7+ times per week	☐ beginning of pregnancy<br>☐ middle of pregnancy<br>☐ end of pregnancy
**6. Fresh Fruit?**<br><br>☐ **Yes**<br>☐ **No**	☐ bananas<br>☐ apples<br>☐ oranges<br>☐ other:<br>__________	☐ 1-3 times in entire pregnancy<br>☐ 1-3 times per month<br>☐ 1-3 times per week<br>☐ 4-6 times per week<br>☐ 7+ times per week	☐ beginning of pregnancy<br>☐ middle of pregnancy<br>☐ end of pregnancy

During your pregnancy, did you drink…			
	**What kind?**	**How many glasses/cans/bottles?**	**When?**
**1. Water?**<br><br>☐ **Yes**<br>☐ **No**	☐ tap water<br>☐ bottled water<br>☐ home filtered<br>☐ other:<br>___________	☐ 1-3 total in entire pregnancy<br>☐ 1-3 per month<br>☐ 1-3 per week<br>☐ 4-6 per week<br>☐ 7+ per week	☐ beginning of pregnancy<br>☐ middle of pregnancy<br>☐ end of pregnancy
**2. Energy Drinks?**<br><br>☐ **Yes**<br>☐ **No**	☐ Monster<br>☐ Red Bull<br>☐ Rockstar<br>☐ other:<br>___________	☐ 1-3 total in entire pregnancy<br>☐ 1-3 per month<br>☐ 1-3 per week<br>☐ 4-6 per week<br>☐ 7+ per week	☐ beginning of pregnancy<br>☐ middle of pregnancy<br>☐ end of pregnancy
**3. Milk?**<br><br>☐ **Yes**<br>☐ **No**	☐ whole<br>☐ low-fat<br>☐ skim<br>☐ organic<br>☐ other: __________	☐ 1-3 total in entire pregnancy<br>☐ 1-3 per month<br>☐ 1-3 per week<br>☐ 4-6 per week<br>☐ 7+ per week	☐ beginning of pregnancy<br>☐ middle of pregnancy<br>☐ end of pregnancy

**4. Juice?**<br><br>☐ **Yes**<br>☐ **No**	☐ orange<br>☐ apple<br>☐ juice blends<br>☐ other: ___________	☐ 1-3 total in entire pregnancy<br>☐ 1-3 per month<br>☐ 1-3 per week<br>☐ 4-6 per week<br>☐ 7+ per week	☐ beginning of pregnancy<br>☐ middle of pregnancy<br>☐ end of pregnancy
**5. Beer or Wine?**<br><br>☐ **Yes**<br>☐ **No**	☐ beer<br>☐ wine<br>☐ malt beverage<br>☐ other: ___________	☐ 1-3 total in entire pregnancy<br>☐ 1-3 per month<br>☐ 1-3 per week<br>☐ 4-6 per week<br>☐ 7+ per week	☐ beginning of pregnancy<br>☐ middle of pregnancy<br>☐ end of pregnancy
**6. Coffee, tea, or soda?**<br><br>☐ **Yes**<br>☐ **No**	☐ coffee<br>☐ tea<br>☐ soda<br>☐ decaf beverages<br>☐ other: ___________	☐ 1-3 total in entire pregnancy<br>☐ 1-3 per month<br>☐ 1-3 per week<br>☐ 4-6 per week<br>☐ 7+ per week	☐ beginning of pregnancy<br>☐ middle of pregnancy<br>☐ end of pregnancy
**7. Mixed drinks or liquor?**<br><br>☐ **Yes**<br>☐ **No**	☐ mixed drinks<br>☐ liquor/shots<br>☐ other: ___________	☐ 1-3 total in entire pregnancy<br>☐ 1-3 per month<br>☐ 1-3 per week<br>☐ 4-6 per week<br>☐ 7+ per week	☐ beginning of pregnancy<br>☐ middle of pregnancy<br>☐ end of pregnancy

**During your pregnancy, did you take…**			
	**What kind?**	**How often?**	**When?**
**1. Over-the-counter medications?**<br><br>☐ **Yes**<br>☐ **No**	☐ sinus decongestants<br>☐ cough/cold syrup<br>☐ ibuprofen (Advil)<br>☐ aspirin<br>☐ acetaminophen (Tylenol)<br>☐ other:___________	☐ 1-3 times in entire pregnancy<br>☐ 1-3 times per month<br>☐ 1-3 times per week<br>☐ 4-6 times per week<br>☐ 7+ times per week	☐ beginning of pregnancy<br>☐ middle of pregnancy<br>☐ end of pregnancy
**2. Prescription medications?**<br><br>☐ **Yes**<br>☐ **No**	☐ prenatal vitamins<br>☐ antidepressants<br>☐ morning sickness meds<br>☐ pain medication<br>☐ other:___________	☐ 1-3 times in entire pregnancy<br>☐ 1-3 times per month<br>☐ 1-3 times per week<br>☐ 4-6 times per week<br>☐ 7+ times per week	☐ beginning of pregnancy<br>☐ middle of pregnancy<br>☐ end of pregnancy

General Information about yourself: Please check the best response.

What is your age?

☐ **[18-20]** ☐ [21-25] ☐ [26-29] ☐ [30-35] ☐ [36-39] ☐ [40+]

What is your ethnicity?

☐ **White** ☐ Hispanic ☐ African-American ☐ Asian/Pacific Islander ☐ Middle Eastern

What is your highest level of education?

☐ **Elementary School** ☐ Middle School ☐ High School ☐ Some College ☐ College Degree ☐ Graduate Degree

What is your household income?

☐ **$0-$25,000** ☐ $25,001-$50,000 ☐ $50,001-$75,000 ☐ $75,001 - $100,000 ☐ $100,000+

How far along were you when you found out you were pregnant (what month)?

☐**1st** ☐2nd ☐3rd ☐4th ☐5th ☐6th ☐7th ☐8th ☐9th

How many weeks pregnant are you now? Or how many weeks postpartum?__________________________________**.**
